# A specialist’s audit of aggregated occurrence records

**DOI:** 10.3897/zookeys.293.5111

**Published:** 2013-04-19

**Authors:** Robert Mesibov

**Affiliations:** 1 Queen Victoria Museum and Art Gallery, Launceston, Tasmania, Australia 7250

**Keywords:** Millipede, Diplopoda, Australia, occurrence records, data quality, data cleaning, ALA, GBIF

## Abstract

Occurrence records for named, native Australian millipedes from the Global Biodiversity Information Facility (GBIF) and the Atlas of Living Australia (ALA) were compared with the same records from the *Millipedes of Australia* (MoA) website, compiled independently by the author. The comparison revealed some previously unnoticed errors in MoA, and a much larger number of errors and other problems in the aggregated datasets. Errors have been corrected in MoA and in some data providers’ databases, but will remain in GBIF and ALA until data providers have supplied updates to these aggregators. An audit by a specialist volunteer, as reported here, is not a common occurrence. It is suggested that aggregators should do more, or more effective, data checking and should query data providers when possible errors are detected, rather than simply disclaim responsibility for aggregated content.

## Introduction

There are currently three online collections of occurrence records for native Australian millipedes. Two are in the aggregated datasets compiled by the Global Biodiversity Information Facility (GBIF; http://www.gbif.org/) and the Atlas of Living Australia (ALA; http://www.ala.org.au/). The third source is *Millipedes of Australia* (MoA; http://www.polydesmida.info/millipedesofaustralia/), a website built and maintained by the present author, who is a millipede specialist. (For more on MoA, see the Methods section below.)

All three online datasets contain errors. I try to minimise the number of errors in MoA by keeping the taxonomy of the recorded millipedes up to date, by checking latitude and longitude, by using simple digital tools to identify duplicate records and inconsistencies, and by excluding the small number of doubtful records (such as those for museum specimens in taxonomically difficult groups with no recorded identifier).

The error-correcting process for MoA has been a cooperative undertaking with the museums from which MoA gets most of its data. In return for a ‘snapshot’ table of Australian millipede records on a particular date, including all relevant database fields, I audit data items, suggest corrections and query any conflicting or uncertain entries. With the help of museum staff, problems are usually resolved, and both the museum database and MoA benefit from improved data quality.

The process just outlined has been a two-way conversation in which museum collection databases are informally audited by a taxon specialist acting as an *amicus musei*. The conversation between museums, GBIF and ALA is more formal and one-way. Most Australian fauna records have been delivered to these aggregators through an intermediary, the Online Zoological Collections of Australian Museums (OZCAM; http://www.ozcam.org.au/), which is now managed and supported by ALA. Delivery is in the form of records translated from the original museum format into a standard schema developed by the Faunal Collections Informatics Group of the Council of Heads of Australian Faunal Collections (http://www.chafc.org.au/fcig/).

The translated records have been accepted ‘as is’ by the aggregators. Data quality has been the responsibility of the data providers, and the aggregators have warned their users accordingly in general disclaimers:

GBIF: ‘The quality and completeness of data cannot be guaranteed. Users employ these data at their own risk.’ (http://data.gbif.org/terms.htm)

ALA: ‘The Atlas makes the Atlas website and Content available on the understanding that you use them at your own risk – they are provided ‘as is’ and ‘as available’ and you exercise your own skill, judgement and care with respect to their use or your reliance on them.

‘The Atlas and data providers give no warranty regarding the quality, accuracy, completeness, currency, relevance or suitability for any particular purpose of the Content or the Atlas website.’ (http://www.ala.org.au/about-the-atlas/terms-of-use/)

I recently went directly to GBIF and ALA in search of new records for MoA. The search developed into an audit which revealed some previously unnoticed errors in MoA, and a much larger number of errors and other problems in the aggregated datasets. In this paper I report on that audit, discuss some of the data quality problems associated with aggregated occurrence records, and suggest ways in which the conversation between data providers and aggregators can be improved.

## Methods

### GBIF and ALA

I queried GBIF for ‘Diplopoda’ from ‘Australia’ and ALA for ‘Diplopoda’ on 23 December 2012 and downloaded the two text files of records. Sorting and tallying of records were done using a spreadsheet program with assistance from Linux command line tools (awk, comm, sort, sqlite3, uniq). Interested readers can contact me for details of the particular procedures I used, but the audit was straightforward (see Results) and could easily be done using other software. The Appendix to this paper contains the original downloaded GBIF and ALA files and two working files from the audit.

In the 23 December 2012 downloads, GBIF and ALA each held occurrence records from 10 data providers, although not the same 10. To avoid unnecessarily drawing attention to particular providers, I refer to them in this paper as ‘provider A,’ ‘provider B’, etc. Provider names are also partly obscured in the two working files in the Appendix, where records are identified by their unique GBIF or ALA identification numbers rather than their source.

### MoA

MoA started in 2007 as a catalogue of species, with annotated synonymies of genus and species names and details of all known types. I added an occurrence records page for named natives (http://www.polydesmida.info/millipedesofaustralia/localities.html) in early 2012. Users can download records for individual genera either as CSV files or (in abbreviated form) as KML files, both made available with a Creative Commons license (attribution + non-commercial, by-nc). Each CSV and KML file is date-stamped in the file name; files are updated and renamed as I become aware of new records or make minor revisions to old ones. The CSV files can also be downloaded as a group from the records directory (http://www.polydesmida.info/millipedesofaustralia/records/).

I compiled the species occurrence records from the taxonomic literature, museum collection databases, and my own records of specimens that have been deposited in museums but not yet registered. Many of the data items from museum databases were corrected or annotated and most were re-formatted, making the MoA dataset a substantial re-working of information from those sources. Significant amendments were reported to staff at cooperating museums, who generously assisted in clarifying or correcting details of the records (see Introduction). The 23 fields in each CSV are genus; subgenus; species; subspecies; number of specimens (sometimes given separately for males, females and juveniles); identifier; repository; registration (catalogue) number; type status; specimen notes; locality in words; state or territory (within Australia); latitude and longitude (separate fields, in decimal degrees based on WGS84); spatial uncertainty; source of the spatial data; elevation; day, month and year of collection (separate fields); collector; collection notes; and source of the record. For more details see the MoA localities metadata page (http://www.polydesmida.info/millipedesofaustralia/metadata.html).

### Audit limitations

The aim of the audit reported here was not to check every data item in the GBIF and ALA record sets. For many users (including the present author), the most important occurrence records are those for specimens identified to species, and the most important data items are *species names*, *latitude and longitude* and *date of collection* – in other words, where and when a particular species occurred, as evidenced by a specimen lot. The MoA dataset only includes records for named, native Australian species, and currently lacks records for millipedes in the small subclass Penicillata (pincushion millipedes). For purposes of comparison with MoA, the GBIF and ALA datasets were therefore progressively trimmed (see Results) to records for named Australian natives in the other millipede subclass, Chilognatha.

### Latitude and longitude comparisons

**Figure 1.**
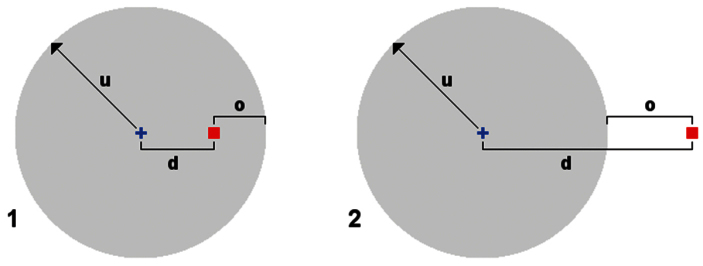
Illustration of ‘uncertainty’, ‘distance’ and ‘offset’. In MoA, spatial uncertainty is defined using the point-radius method, where a site is assumed to be at the centre of a circle whose radius is the uncertainty **u**. In both diagrams, **d** is the Euclidean distance between the MoA estimate of the site’s location (blue cross) and the aggregator estimate (red square). The offset **o** is the distance **d** minus the uncertainty **u**. In diagram **1** the aggregator site is within the circle of uncertainty surrounding the MoA site, and the offset is negative. In diagram **2** the aggregator site is outside the circle of uncertainty and the offset is positive

To compare latitude and longitude data for the corresponding records in MoA and GBIF or ALA, I calculated the Euclidean distance between the two reported locations. I assumed 111 km per degree of latitude, and 111 km times the cosine of the latitude per degree of longitude. I used Euclidean distance because the purpose of this comparison was to detect substantial differences, not to accurately determine the great-circle distance between corresponding MoA and aggregator localities. From this distance I then subtracted the uncertainty estimate included with every georeferenced MoA record. This difference is here called the ‘offset’ between MoA and aggregator latitude and longitude data (Fig. 1). The MoA uncertainty is the radius of a circle likely to contain the collection site (for more details see the MoA metadata page, http://www.polydesmida.info/millipedesofaustralia/metadata.html). I estimated this figure conservatively when compiling MoA; it ranges from 25 m (for most GPS data) to 200 km (e.g., for ‘Kimberley district, Western Australia’). Distance and uncertainty were both rounded in this study to the nearest 1 km, so that the minimum difference between the two would be larger than any Australian datum difference (e.g., between AGD66 and GDA94).

A negative or zero offset meant that the aggregator location, although different from the MoA location, was within my uncertainty estimate (Fig. 1). Whatever the reason for the difference, I could be satisfied that a GBIF or ALA user was seeing an acceptably approximate latitude and longitude for the millipede collecting locality. A positive difference meant that the aggregator location was substantially set off from the location I had compiled in MoA, and the difference needed to be examined more closely (see Results). To reduce the number of records to be individually checked, I examined only those cases where the offset was 2 km or greater.

## Results

### Preparation: overview and minor exclusions

The GBIF dataset for Australian Diplopoda contained 5558 records and the ALA Diplopoda dataset 8690 records, with 4860 records shared and 9554 records in total.

**Figure 2.**
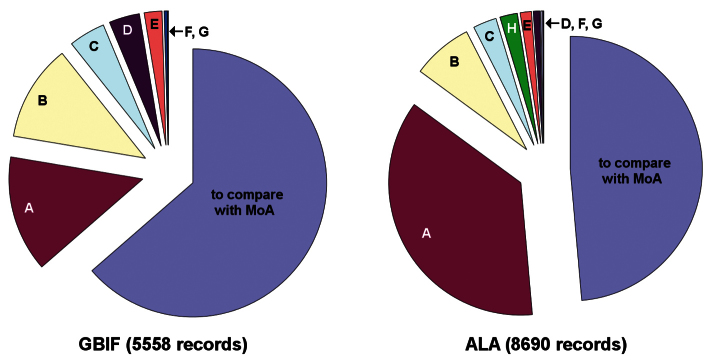
Exclusions from the GBIF and ALA datasets (see text for details). **A** not identified to species or only tentatively identified to species **B** undescribed species **C** non-native species **D** no latitude and longitude **E** manuscript names **F** miscellaneous duplicates **G** Penicillata **H** ALA preliminary exclusions (not in Australia, images, provider K observations). **D** and **F** categories do not include records already excluded for taxonomic reasons

ALA Diplopoda included 111 records from outside continental Australia and its territories. Fifteen ALA records were of millipede images; only three of these were for a named Australian native species, and I could not confirm that species’ identity from the images. These 126 records were excluded from the audit (Fig. 2).

Another 60 ALA records were ‘observations’ of Tasmanian millipedes from provider K. Forty-one of these records were excluded because the sighted millipedes were identified only to genus. Eighteen of the remaining records were unconfirmed species identifications by non-specialists, including two identifications of a species which was not named and described until eight years after the observations. The nineteenth species-sighting record and one of the genus-sightings were attributed to me, but I have never knowingly contributed records to provider K. Several of the 60 ‘observations’ appeared to be duplicates of specimen records in a Tasmanian museum, but I could not be sure because the locations, dates and collector names differed in detail from the museum records. The 60 ALA records from provider K were excluded from the audit (Fig. 2).

Two data providers created minor bookkeeping issues. Provider J supplied GBIF with 75 records for specimen lots from three museums, but rather than the GBIF *Catalogue number* field being filled with the museums’ catalogue numbers, the entries were instead provider J’s own internal system numbers for the records. Provider B neglected to enter the catalogue number for a particular specimen lot; the *Catalog Number* field remains blank in the ALA dataset, but confusingly has been filled in the GBIF *Catalogue number* field with the ALA *Record ID* code. (The correct catalogue number was supplied to me for MoA use by provider B.)

### Preparation: taxonomic exclusions

I excluded from the remaining GBIF and ALA records any which were not for named, native species in the suborder Chilognatha (Fig. 2). These taxonomic exclusions were for millipedes that were:

(a) identified only to class (640 records);

(b) identified only to order (2708 records);

(c) identified only to family (427 records);

(d) identified only to genus (88 records);

(e) identified as a non-native species (251 records);

(f) identified as a species of Penicillata (9 records);

(g) identified with unpublished names (manuscript or museum-label names), e.g. *Subarricrusta biconulata* (119 records);

(h) identified as an undescribed species in a named genus, e.g. *Myallosoma* ‘wagga’ (289 records);

(i) identified as an undescribed species in an undescribed genus, e.g. Genus QYY sp. QYY3 (361 records); and

(j) identified only tentatively to species, e.g. ‘*Antichiropus variabilis*?’ (8 records).

### Preparation: latitude and longitude exclusions

I excluded 212 records without latitude and longitude (Fig. 2). (Some records excluded earlier in the trimming process also lacked latitude and longitude.)

### Preparation: excluding duplicates

The purpose of the GBIF and ALA audit was to check occurrence records which placed a particular species at a particular place at a particular time. This information might be repeated in a record set in a number of ways:

(a) ‘Simple’ duplicates repeat the record for a particular specimen lot in a particular repository. In the ALA dataset, provider H had two different names as data provider, and the same specimen lot appears under each provider name. In the GBIF dataset, provider J (a provider of records from a range of specimen repositories and the taxonomic literature) supplied a record identical to one from provider E. (4 simple duplicates, already excluded for other reasons)

(b) ‘Satellite’ duplicates are created when material is removed from a specimen for SEM work, DNA extraction, etc. Provider A gave the new collection object the same catalogue number as the source object, but with a suffix appended, e.g. ‘X43302’ and ‘X43302.001’. (31 records, 27 already excluded)

(c) ‘Bookkeeping’ duplicates appear when a museum specimen lot is renumbered or donated to another museum, but the original catalogue number is not cleared of data. Another kind of bookkeeping duplicate appeared in the dataset supplied to GBIF by provider J: the only difference between paired duplicates was that the GBIF *Basis of record* field contained either ‘Specimen’ or ‘Unknown’. Two records from provider B contained incorrect data, according to advice I had from provider B in 2012; their continued presence in the GBIF and ALA datasets is a bookkeeping issue. These two records have the same catalogue number as other specimen lots, but different suffixes. (60 records, 52 already excluded)

(d) ‘Serial’ duplicates appear when multiple catalogue numbers are assigned to a series of specimens of a single species arising from the same collection event, i.e. same species, same site, same collector, same date. The multiple catalogue numbers may refer to a single specimen lot; provider F, for example, puts multiple labels into individual glass vials, one for each millipede specimen in the vial. Alternatively, a specimen lot may be physically divided, e.g. into holotype and paratype lots, or male and female lots, with each lot being assigned its own catalogue number. Provider B assigns a single catalogue number to each specimen lot, but adds a suffix to distinguish male and female components of the sample.

I excluded all simple, satellite and bookkeeping duplicates from the audit, but included all serial duplicates except nine ‘suffixed’ provider B records (Fig. 2). Including serial duplicates simplified the comparison with MoA records, nearly all of which are uniquely identified by a museum catalogue number.

### Preparation: summary

The final tallies of records for the audit were 3536 in the GBIF dataset (64% of the starting number), 4223 in the ALA dataset following the 186 preliminary exclusions (50%), 3524 records shared and 4235 records in total (Fig. 2). (The corresponding total for MoA was 10615 records.)

## Comparison: introduction

One GBIF-ALA record was in the MoA dataset, but with a different provider G catalogue number; the specimen lot had been renumbered but the provider G database had not yet been updated. Thirty-five GBIF-ALA records were missing from the MoA dataset, for a variety of reasons:

(a) 3 provider A records I overlooked when compiling MoA;

(b) 7 provider I records and 1 provider A record were not in the provider databases at the time the MoA dataset was first compiled, in 2011-2012;

(c) 7 provider D records were not publicly available before they were published by GBIF;

(d) 5 provider G records are for specimen lots donated to other museums (whose records are not in GBIF or ALA), but not yet cleared from the provider G database;

(e) 4 provider G records for specimen lots identified only to genus are incorrectly listed as identified to species;

(f) 1 provider G record is for a blank catalogue number, incorrectly filled with data;

(g) 4 provider A records I excluded from MoA because the species concerned are in taxonomically difficult groups and no identifier was listed in the provider A database for those records;

(h) 2 provider A records I excluded because the identifications were clearly incorrect, one being a ‘Paradoxosomatidae’ specimen (Polydesmida) from Western Australia identified as a species of Spirobolida endemic to Lord Howe Island; and

(i) 1 provider A record I excluded because I judged the locality (‘Tasmania’) to be too vague to be georeferenced; provider A has georeferenced as ‘Tasmania’ a point near the north coast of the island.

For comparison with MoA, I added to the matching MoA dataset the (d), (e) and (g) records, and the above-mentioned, renumbered record from provider G, which was incorrectly listed as identified to species.

### Comparison: species names

In addition to auditing species names, I checked the advice relating to names which is included with each ALA record. ALA assists users by comparing submitted names to those on a relevant National Species List (NSL). ALA’s list of millipede names is derived from the Australian Faunal Directory (AFD; http://www.environment.gov.au/biodiversity/abrs/online-resources/fauna/index.html), whose millipede section I updated in 2010.

ALA flags possible name problems in two ways. First, it attempts to match the provided name with one in the NSL, and if there is no match it offers – in the *Matched Scientific Name* field – a name in the lowest ranking taxonomic category above species which might apply to the provided name, e.g. a genus name. Second, it enters ‘true’ or ‘false’ in the field *Name not in national checklists*, where ‘true’ means that the name is not in the NSLs, but is listed in Catalogue of Life (CoL; http://www.catalogueoflife.org/). However, as shown below, name-matching was inconsistently effective as a check on data quality in the millipede dataset.

(a) Provider G supplied 67 records with the wrong species names, i.e. incorrect specimen identifications. I supplied correct identifications for these records in 2005, but provider G’s database has not yet been updated. ALA accepted three of the names ‘as is’, since they were correctly spelled and in the NSL. ALA correctly matched the new genus combination applicable to the other 64 incorrect species names.

(b) Eight records had misspelled species names. ALA did not recognise *Hoplatessara pugonia* (= *Hoplatessara pugiona*), *Myallosoma furculigerium* (= *Myallosoma furculigerum*) and *Reginaterreuma tarkensis* (= *Reginaterreuma tarkinensis*; two records), and matched these four names with the (correct) genus. The misspelled *Australiosoma ehteridgei* is now *Dicladosoma etheridgei*; ALA matched the record with the genus *Australiosoma*. Three records for *Siphonophora mjohergi* (= *Rhinosiphora mjoebergi*) were matched with the family Siphonophoridae.

(c) Two records had the misspelled genus name *Aethelosoma* for *Aethalosoma*. In this case ALA matched the two *Aethalosoma solum* records with the correctly spelled genus (but not the species name), suggesting that fuzzy matching (or manual editing) had been applied.

(d) One record had *Tasmaniosoma hardyi* for *Tasmanodesmus hardyi*. ALA incorrectly matched this species with the genus *Tasmaniosoma*.

(e) Data providers submitted 157 records with outdated genus combinations, several of which are noted above. For 141 of these records ALA matched the outdated combination with its correct NSL combination. Five matchings were thwarted by incorrectly spelled species names (see above). *Cyliosoma excavatum* (now *Epicyliosoma excavatum*), *Cyliosoma penrithensis* (*Epicyliosoma penrithense*, five records), *Cyliosoma penicilligerum* (*Epicyliosoma penicilligerum*) and *Spirobolus lugubris* (*Spirobolellus lugubris*) were matched with the older combinations in CoL, although all names are in the AFD. The AFD also lists *Dicladosoma andersoni* (*Phyllocladosoma andersoni*), which was matched with *Dicladosoma*, and *Phyllocladosoma andersoni dorrigense* (*Phyllocladosoma dorrigense*, two records), which was matched with *Phyllocladosoma*.

(f) Data providers submitted 14 records with two outdated synonyms of *Cladethosoma trilineatum*, one of *Akamptogonus novarae* and one of *Parwalesoma walesium*. ALA matched synonyms correctly in eight of the cases. In the other six records *Cladethosoma clarum* was accepted, although that synonym is referred to *Cladethosoma trilineatum* in the AFD.

(g) The species *Prosopodesmus panporus* (two records) was matched with the genus *Prosopodesmus*, while both *Prosopodesmus crater* and *Prosopodesmus monteithi* were matched with their correct species names. All three names are listed in the AFD and all *Prosopodesmus* records were contributed by provider B.

(h) Finally, ALA accepted *Antichiropus variabilis ingens* (two records), apparently because the name is in CoL; the *Name not in national checklists* field has the entry ‘true’. The subspecies name was suppressed in 1920 and does not appear in the AFD.

In all, 174 records had species names different from the correct species names in MoA, and another 10 records not in MoA also had incorrect or outdated names.

### Comparison: latitude and longitude - overview and results

A trial comparison revealed that there were bookkeeping discrepancies in four MoA records from provider F. In two cases, two consecutive catalogue numbers and their collecting data in MoA had been exchanged, as compared to the entries in provider F’s collection database. I hope to investigate the discrepancy on my next visit to provider F, later in 2013, but for the purposes of this comparison the four MoA records were renumbered to agree with those of provider F.

Following that renumbering, 1144 of the 4209 records compared (27%) were found to have an offset of 2 km or more and 651 records (15%) had an offset of at least 5 km; 19 records had an offset of 100 km or more. MoA was clearly to blame for 22 discrepancies, because I had:

(a) given provider F the wrong longitude (146°23’13”E instead of 146°28’13”E) for one of my collecting sites (15 records),

(b) incorrectly copied the latitude or longitude from providers’ databases to MoA (1 record),

(c) assigned to a record in MoA the spatial data from the wrong collecting event (3 records),

(d) incorrectly georeferenced a record from label locality text (1 record), and

(e) used georeferences from provider B which provider B did not accept (2 records).

The last two discrepancies arose because I used latitude and longitude data downloaded for the samples concerned from a provider B website. Provider B has recently advised me that if the latitude and longitude are printed on a specimen label, then those figures are accepted for the provider B database. If there are no geographical coordinates on the label, a gazetteer-based georeferencing program is used to calculate a location from the label locality text. In these two cases, provider B (correctly) ignored its own Web-published latitude and longitude data for the sites and calculated new ones for its database, whose records were then reformatted for export to OZCAM. This practice can lead to errors (see below, *Comparison: latitude and longitude - comments*).

The largest single explanation for the discrepancies, resulting in (at least) 968 incorrect latitude and longitude figures, was a decision made by provider G in the 1990s. When collectors supplied UTM grid references for collecting sites, these grid references were duly entered in UTM fields in the provider G database. However, to populate the latitude and longitude fields for those records, provider G chose not to convert directly from UTM. Instead, the data enterer at provider G would search in a lookup table for a ‘nearest named place’ (NNP) close to the place named in the locality text, then enter the latitude and longitude for that NNP. This practice resulted in three kinds of spatial errors:

(a) The data enterer chose the wrong NNP. For example, searching for ‘Christmas Hill’ in the lookup table, the data enterer selected ‘Christmas Hills’, 100+ km distant.

(b) The NNP was a substantial distance from the actual collecting site and was differently named. For example, the site was at River O’Plain Creek near English Town, and the NNP chosen was English Town.

(c) The NNP text and the locality text agreed, but the actual collecting site was a substantial distance from the location listed in the lookup table. For example, the site was identified as ‘Mersey River’, but the NNP for ‘Mersey River’ was at a different point on the same stream.

The great majority of the UTM grid references for these records had spatial uncertainties of 100 m. By replacing these grid references with latitude and longitude to the nearest minute, provider G not only misplaced the collecting sites, but increased their spatial uncertainties more than 10-fold. Provider G no longer replaces grid-referenced locations with NNP locations, but has not yet corrected the records created when the NNP policy was in place.

The remaining latitude and longitude discrepancies of 2 km or more had other explanations:

(a) The aggregator latitude and longitude had been rounded off, e.g. -36.9667 147.15 in the provider database became -37 147.2 (40 records). Provider I accounted for 38 of these discrepancies, in all cases because the species concerned was on a protected species list, and localities were partly disguised by rounding off (see below, *Comparison: latitude and longitude - comments*). On enquiry, provider F could not suggest why two of its latitude and longitude figures had been rounded off after uploading to OZCAM.

(b) The latitude and longitude were determined from locality text naming a large place (e.g. ‘Fraser Island’), and the discrepancy arose because the provider and the MoA spatial data source chose different georeference points in the place (16 records).

(c) As for (b) but the place was only vaguely named, e.g. ‘Upper Richmond River’, the name of a district in New South Wales in the 1890s (17 records).

(d) The locality text on the label (used in MoA) was more exact than the text used for georeferencing in the provider database, e.g. ‘Blue Mountains - Katoomba - Echo Point’ on the label vs. ‘Blue Mountains’ in provider A’s database (25 records).

(e) The provider incorrectly georeferenced the collection site from locality text (47 records). In 11 of these cases, the provider subsequently corrected both the provider’s database and MoA with an improved latitude and longitude.

(f) The provider entered spatial data into its database incorrectly (3 records). One of the discrepancies resulted from a simple transcription error, while in the other two cases a specimen lot was assigned to the wrong collecting event.

(g) The provider and I subsequently found that we had both incorrectly georeferenced the collecting site, and we agreed on an improved latitude and longitude for use by the provider and MoA (2 records).

Finally, four of the 1144 discrepancies remain unresolved at the time of writing, and will be further investigated by myself and the data providers concerned. In two cases it is possible that there has been a bookkeeping error like the one described at the beginning of this section. In the other two cases there is a puzzling disagreement between the locality text and the latitude and longitude provided by the collector.

### Comparison: latitude and longitude - comments

A surprising feature of the aggregated occurrence records is that locality text is not always included. Only 1908 of the 4235 records used for comparison (45%) have locality text in the *locality* (ALA) or *Locality* (GBIF) fields, although ‘locality’ is a recommended field in the OZCAM schema. Nearly all of the 2000+ records without locality text in the GBIF and ALA datasets include that text in the relevant data provider’s databases. (All MoA records include locality text.)

I was unable to see any fields in the ALA download which would alert users to possible georeferencing problems, other than a *Coordinates don’t match supplied state* field. The download has a *Location Quality* field with the entries ‘Spatially suspect’ and ‘Spatially valid’. These values are not explained on the website to which ALA users are directed for more information (https://docs.google.com/spreadsheet/ccc?key=0AjNtzhUIIHeNdHhtcFVSM09qZ3c3N3ItUnBBc09TbHc#gid=0), and I was unable to see how the values related to the records. A series of ALA records with exactly the same spatial data (serial duplicates), might have both ‘suspect’ and ‘valid’ in the *Location Quality* field.

Equally puzzling are the ALA flagging fields *missing Coordinate Precision* and *Coordinate uncertainty not specified*. In the full 8690-record ALA download, there are no records at all with entries in the *Coordinate Precision* field, yet 121 of those records have ‘false’ instead of ‘true’ for *missing Coordinate Precision*. The *Coordinate Uncertainty in Metres - parsed* field is populated with numbers in 1728 records, yet for five of those records *Coordinate uncertainty not specified* reads ‘true’, and for 121 records without a parsed uncertainty entry, *Coordinate uncertainty not specified* reads ‘false’.

It was curious to find that ALA and GBIF (and presumably OZCAM) had accepted georeferences to large numbers of decimal places, e.g. -17.6000003814697 145.699996948242 for a locality the collectors described in 1971 as ‘ca 12 km SE of Millaa Millaa’ (Queensland). The 13th decimal place locates the site with sub-micron accuracy, and the latitude and longitude could be simplified to -17.6 145.7 with no significant loss of precision. All MoA latitude and longitude data are compiled to four decimal places, which in Australia corresponds to ca 8-11 m on the ground. The implied spatial uncertainty of ca ±4-5 m is equal to or smaller than the error in most handheld GPS readings (Mesibov 2012).

Although there is an excellent, freely available guide (Chapman and Wieczorek 2006) to georeferencing from locality text, there is scope for disagreement between practitioners, as in the ‘vague locality’ cases above, about both location and spatial uncertainty. MoA and the aggregators will continue to disagree in these instances, and also with regard to latitude and longitude for protected Western Australian millipedes (see above). Disguising protected species localities in aggregated sources by rounding off latitude and longitude is appealing as a conservation measure. However, more accurate latitude and longitude figures for the same sites are often readily available online in digitised taxonomic literature and consultants’ reports, so the disguise affects only those users who only consult aggregators.

As noted above, provider B uses a gazetteer-based georeferencing program when specimen labels lack latitude and longitude. I found a number of records for which this policy had resulted in an incorrect location. Two examples are worth examining in detail to demonstrate how the policy has been implemented:

(a) In 1990 and 1991, a number of Australian entomologists collected specimens near Pelion Hut, in Tasmania’s Cradle Mountain National Park. Specimen labels were printed for most samples with the correct latitude and longitude, namely 41°50'S, 146°03'E. A 1991 Malaise trapping by one of the collectors also included samples from ‘Pelion Hut’, but no latitude and longitude was printed on the specimen labels. Provider B queried its georeferencing program, which located Pelion Hut at 41°50'S, 146°05'E, 3 km to the east. After alerting provider B to the problem, I contacted the collector for more information. He confirmed that his 1991 trap sites were all close to the Overland Track, which runs past Pelion Hut, and that he did not sample 3 km east of Pelion Hut. I passed this information on to provider B. (1 record in the trimmed GBIF and ALA datasets)

(b) In November 1982, a field worker collected a series of samples in the Mt Royal Range in New South Wales. The collecting sites were located as road distance from the village of Moonan Flat, which lies west of the Range, e.g. ‘Gologolie Creek, 17 km E of Moonan Flat’, ‘Horse Swamp, 24 km E of Moonan Flat’ and ‘Cobark Camp, 49 km E of Moonan Flat’. Another site was recorded as ‘Mt Royal Range, Devils Hole, 36 km W of Moonan Flat’. The road distance to the Devils Hole camping ground is now approximately 32 km, and it is clear that ‘W’ is an error for ‘E’. Because there were no latitude and longitude figures on the specimen labels for the Mt Royal Range collections, provider B queried its georeferencing program. In this case, provider B georeferenced ‘36 km W of Moonan Flat’, ca 60 km west of Devils Hole, and ignored the locality text ‘Mt Royal Range, Devils Hole’. According to a colleague, the specimen labels for this site also mention *Nothofagus* forest (temperate rainforest), which occurs in the Mt Royal Range but not in the dry country west of Moonan Flat. Provider B has now edited the latitude and longitude for this site in its own database to 31°55'S, 151°36'E for ‘36 km E of Moonan Flat’ in the revised locality text. This is ca 10 km east of Devils Hole, whose correct latitude and longitude is 31°55'S, 151°29' E. (3 records in the full GBIF and ALA datasets)

### Comparison: collecting dates

The combined GBIF and ALA datasets included collecting dates for all but 160 records; MoA had dates for 94 of these, taken from provider databases or specimen labels.

There were 384 date discrepancies in the comparison, but 274 of these were due to a difference in the way collecting periods were recorded. MoA had the finish date of a period in the day, month and year fields, and the whole of the period (e.g. ‘15 Mar - 6 Apr 1988’) in the collecting notes field. The OZCAM schema requests a single date, and providers generally supplied the start date for a period (in 10 cases, a date in the middle of the period). The remaining discrepancies arose because:

(a) I entered the MoA date incorrectly, or assigned the wrong collecting event to the specimen lot (29 records);

(b) I used for MoA the date on the specimen label, which disagreed with the provider database date, e.g. ‘9 November 1970’ (label) vs ‘November 1970’ (database) (3 records);

(c) The provider entered the wrong date in the database (46 records);

(d) ALA or provider I changed a month-year date to the last preceding day-month-year date; e.g. July 2003 became 30 June 2003 (20 records); or

(e) The date was uncertain and still needs to be checked (10 records).

The last two date discrepancies are between MoA and ALA, on the one hand, and GBIF, on the other. For unknown reasons GBIF has 27 April 1976 instead of 27 October 2005 for one record, and 10 September 2001 instead of 10 September 2004 for the other. The localities and collectors for these two records in GBIF are correct.

## Discussion

### Outcomes

I contacted six of the MoA data providers with suggestions for database corrections arising from the audit, and with requests for additional information. One of those requests led to museum staff discovering errors in two records recently uploaded to OZCAM but not yet added to MoA, and a number of the requests encouraged providers to revisit locality data and improve latitude and longitude figures for both their own databases and MoA. The latter was considerably improved as a result of the audit and the follow-up contacts: I added 11 occurrence records, deleted seven and corrected 95.

MoA has been updated, but it is uncertain when recent corrections to museum databases (following both the MoA compilation audit in 2012 and the audit described above) will be reflected in GBIF and ALA. The error-correction flow is

specialist → data providers → OZCAM → GBIF, ALA

and if I had contacted the aggregators directly, presumably

specialist → GBIF, ALA → data providers → OZCAM → GBIF, ALA

Aggregated datasets are only periodically updated, with data providers supplying edits and new records asynchronously. It will be some time before the many errors noted above in provider G’s database will be corrected, although I have been advised that the database software currently used by provider G allows for easier record editing than was previously possible, and the millipede records, at least, should be up to date before the end of 2013.

### Feedback

It is sometimes argued that a potential benefit of aggregating biodiversity datasets and putting them online is that errors are exposed to a larger audience than would be the case if records only appeared on individual museum websites, or were not otherwise publicly available. I write ‘potential’ because it is unclear whether non-specialist users would have the time or interest to audit aggregated, abbreviated records in the way I did for the unabbreviated records that are the basis for the MoA dataset. As noted above in the latitude and longitude comparison, a majority of the aggregated records lacked the locality text included in the data provider’s own databases. Exposing records to a wide audience online may be a good way to crowd-source data quality checks, but only if the crowd gets to see the information it needs.

In any case, there do not seem to be established, well-used pathways for information about errors or possible errors to get back directly to data providers from the aggregators. Queries arising from the checking of species names done by ALA (see Results) may not be routinely passed on to the institutions concerned, although they should be. Certainly questions about species names not on the NSLs could be asked, although data providers may need to consult specialists to get answers. As shown above, automated name-checking did not always work, but could be improved with a fuzzy-matching algorithm.

Checking procedures for other data problems may be even less effective. None of the duplicate records I found was flagged as ‘true’ in the ALA field ‘Inferred Duplicate Record’, or flagged in any way by GBIF despite recent advances in duplicate detection by that organisation (GBIF 2012). Georeferencing checks were minimal (see Results). The aggregators could explore the approach used in MoA, which is to define collecting events based on the union of data for locality, collector(s) and date. In addition to checks of the separate data items, checks of grouped events can reveal otherwise obscure errors; for an example, see the ‘Moonan Flat’ case in *Comparison: latitude and longitude - comments*, above.

The MoA occurrence records are freely available for use by aggregators. However, the MoA dataset is in large part derived from records provided by the same institutions that supply GBIF and ALA. It is unclear how aggregators would deal with two versions of the same record – one as received from a data provider, and one as amended in MoA – or with the attendant licensing issues. A possible solution would be for an aggregator to use the MoA dataset in data quality checks, as ALA uses National Species Lists, and to query data providers and the present author with regard to discrepancies. Since aggregators seem to prefer flagging possible errors to communicating with their data sources, it is unlikely that MoA will be used this way.

### Value of aggregated data

Whether or not new checks are implemented by the aggregators and communicated to their data providers, the body of aggregated millipede data is likely to remain a curate’s egg (http://en.wikipedia.org/wiki/Curate%27s_egg). Providers F and I only upload records of named millipede species to OZCAM, while provider A uploads everything in its database classified under ‘Diplopoda’. Provider G has been through two changes of collection database software in the last 10 years, and most of its records have not been checked or updated in that time. The unused sighting records from ALA’s provider K (see Results, *Preparation: overview and minor exclusions*) are not unique in their problems. In recent correspondence with that provider, I was told that a dataset for another taxon had been corrupted by unskilled data entry in 2007 and a problem with dates in Microsoft Excel (http://support.microsoft.com/kb/180162), which shifted observation dates by four years and one day. My contact told me that provider K had not yet had the time or resources to check its records carefully.

The aggregated occurrence records for Australian millipedes are not only variable in quality but a long way from complete, because not all major sources are aggregated. Three of the Australian museum sources of the 10600+ MoA records are either minimally represented in the GBIF and ALA datasets, or not represented at all.

There is no reason to doubt that staff at both GBIF and ALA are genuinely interested in improving the quality of aggregated data, and both organisations regularly issue advice and discussion documents on improvements to the flagging of problems in records (e.g. ALA undated, GBIF 2011). Nevertheless, like Yesson et al. (2007), I found a fairly high error rate in the aggregated data I audited. Using a conservative spatial error criterion and excluding ‘acceptable’ spatial differences (protected species approximations, large or vague places to be georeferenced), and also excluding differences in the treatment of collecting periods (start date vs finish date of period), roughly one in four records in the combined GBIF-ALA dataset of 4235 records tested against MoA contained at least one error. The GBIF and ALA disclaimers quoted in the Introduction are pertinent warnings to users of aggregated data, and are likely to remain so for some time to come.

## Acknowledgements

I am very grateful to the curators and data managers who have cooperated with me in improving Australian millipede occurrence records. Arthur Chapman, Mark Costello and Rod Page made helpful and constructive comments on a draft of this paper. The audit was voluntarily undertaken by the author.

## Appendix

ALA and GBIF occurrence records. (doi: 10.3897/zookeys.293.5111.app) File format: Archive (zip). **Explanation note:** The Appendix file contains three sets of data. ALA_Diplopoda_23-Dec-2012 and GBIF_Diplopoda_Australia_23-Dec-2012 are CSV files of occurrence records as downloaded on 23 December 2012. Working_files contains two CSV files: (1) Full GBIF and ALA records for Australian millipedes, with annotations for those excluded from the audit (Exclusions_within_full_datasets.csv), and (2) Comparison of GBIF and ALA records with corresponding MoA records, as described in paper (Comparison_table.csv).
