# Supplementary material for: A specialist’s audit of aggregated occurrence records
Source: Zookeys. 2013 Apr 19;(293):1–18. doi: 10.3897/zookeys.293.5111 (PMC3677402; doi:10.3897/zookeys.293.5111)
Supplement: Supplementary file 1 — ALA and GBIF occurrence records. (doi: 10.3897/zookeys.293.5111.app) File format: Archive (zip). Explanation note: The Appendix file contains three sets of data. ALA_Diplopoda_23-Dec-2012 and GBIF_Diplopoda_Australia_23-Dec-2012 are CSV files of occurrence records as downloaded on 23 December 2012. Working_files contains two CSV files: (1) Full GBIF and ALA records for Australian millipedes, with annotations for those excluded from the audit (Exclusions_within_full_datasets.csv), and (2) Comparison of GBIF and ALA records with corresponding MoA records, as described in paper (Comparison_table.csv). [file ZooKeys-293-001-s001.zip › ALA_Diplopoda_23-Dec-2012/README.html]

For more information about the fields that are being downloaded please consult Download Fields.
